# Repeatability of swimming activity of the Patagonian grouper *Acanthistius patachonicus* based on accelerometry

**DOI:** 10.1093/conphys/coae074

**Published:** 2024-10-28

**Authors:** Leonardo A Venerus, Paolo Domenici, Stefano Marras, Lucas E Beltramino, Javier E Ciancio

**Affiliations:** Centro para el Estudio de Sistemas Marinos, Consejo Nacional de Investigaciones Científicas y Técnicas (CESIMAR, CCT CONICET-CENPAT), Blvd. Brown 2915, U9120ACD Puerto Madryn, Chubut, Argentina; Consiglio Nazionale delle Ricerche, Istituto per lo Studio Degli Impatti Antropici e Sostenibilità in Ambiente Marino (CNR-IAS), Località Sa Mardini, 09070 Torregrande, Oristano, Italy; Consiglio Nazionale delle Ricerche, Istituto di Biofisica (CNR-IBF), Area di Ricerca San Cataldo, Via G. Moruzzi 1, 56124 Pisa, Italy; National Biodiversity Future Center (NBFC), Piazza Marina 61, 90133 Palermo, Italy; Consiglio Nazionale delle Ricerche, Istituto per lo Studio Degli Impatti Antropici e Sostenibilità in Ambiente Marino (CNR-IAS), Località Sa Mardini, 09070 Torregrande, Oristano, Italy; National Biodiversity Future Center (NBFC), Piazza Marina 61, 90133 Palermo, Italy; Universidad Nacional de la Patagonia San Juan Bosco (UNPSJB), Blvd. Brown 3051, U9120ACE Puerto Madryn, Chubut, Argentina; Centro para el Estudio de Sistemas Marinos, Consejo Nacional de Investigaciones Científicas y Técnicas (CESIMAR, CCT CONICET-CENPAT), Blvd. Brown 2915, U9120ACD Puerto Madryn, Chubut, Argentina

**Keywords:** Behaviour, free-living marine fishes, Northern Patagonia, rocky-reef fishes, tailbeats, tri-axial accelerometers

## Abstract

The study of repeatability in behaviour and activity level can be used to evaluate inter-individual differences, which are fundamental to assess the resilience of populations to environmental variation. Previous work on repeatability in wild fish populations has largely been based on acoustic telemetry or mark-and-recapture and has revealed repeatable activity patterns over relatively long periods in a number of species. Although accelerometry is a promising tool for investigating the swimming activity of fish in the wild, little is known about the repeatability of accelerometry-based traits in wild fish. Here, we used external accelerometers to investigate the swimming activity of the Patagonian grouper *Acanthistius patachonicus*, a rocky-reef fish with high site fidelity, which ensures a high recapture rate of accelerometer tags. Accelerometry was used to investigate the short-term repeatability of a number of activity traits, including swimming, hovering, daily median tailbeat frequency, percentage of high tailbeat frequency and total number of tailbeats at different times of the year. We found that all of the variables are repeatable over the daily scale and four out of five variables are repeatable over weekly periods. Overall, our work suggests that these traits are individual-specific for the short time period investigated. In addition, the percentage of time spent in swimming and hovering was greater in the warm season compared to the cold season, suggesting higher activity levels related to higher temperatures. These results suggest that activity traits related to swimming are repeatable and likely related to the physiological state of each individual. Finally, our work shows that accelerometry can be considered a valuable tool to explore inter-individual differences with potential applications for assessing the resilience of wild populations.

## Introduction

Swimming activity can vary both amongst and within species (Kolok *et al.*, 1998; Claireaux *et al.*, 2007; Marras *et al.*, 2010). Certain pelagic species such as tuna and clupeids are known to be very active, whilst benthic species tend to be more sedentary (Videler, 1993; Webb, 1994). However, even within a given species, it is now known that activity level can vary amongst individuals and that this is correlated with physiological and behavioural characteristics (Killen *et al.*, 2013). The tendency of an individual to express a certain behaviour or activity level can be investigated by assessing its trait repeatability (Killen *et al.*, 2016). The assessment of repeatability is a fundamental issue in ecology and evolution, because it allows to quantify the extent to which behavioural or physiological traits can be shaped by selection (Hitchcock *et al.*, 2015). For this reason, trait repeatability has been suggested to provide the upper limit for trait heritability (Dochtermann *et al.*, 2015). Within the context of conservation, the level of within-individual variability in behaviour and physiology is related to the vulnerability of individuals that must cope with anthropogenic environmental change (e.g. pollution, climate warming, habitat loss, etc.). Understanding the role of within-individual variation on population responses to the environment is an open area for research (Killen *et al.*, 2016).

In the past couple of decades, there has been a growing interest in the repeatability of traits in fishes, with much work being based on laboratory observations of metabolism, locomotor activity and behavioural traits such as boldness and aggression (Nespolo and Franco, 2007; Williams, 2008; Bell *et al.*, 2009). Repeatability has been defined as the proportion of within-population variation, as opposed to within-individual variation (Biro and Stamps, 2015). A highly repeatable trait therefore is a trait that is consistently repeated in each individual of the population. Although most evidence of trait repeatability in fishes is based on laboratory studies (Nelson and Claireaux, 2005; Oufiero and Garland, 2009; Marras *et al.*, 2010, 2011; Killen *et al.*, 2016) or on studies of individuals who lived at sea between measurements (e.g. Handelsman *et al.*, 2010; Vandamm *et al.*, 2012), there is a growing interest in quantifying repeatability in the field (e.g. Claireaux *et al.*, 2007; Forsythe *et al.*, 2012; Taylor and Cooke, 2014; Harrison *et al.*, 2015; Killen *et al.*, 2016; Alós *et al.*, 2017; Villegas-Ríos *et al.*, 2017; Griffin *et al.*, 2023).

Fieldwork poses a number of challenges that include partitioning the repeatability resulting from environmental effects from the one intrinsic to individual animals (Killen *et al.*, 2016), as well as the technical difficulties of measuring behaviour and activity in the wild. In particular, a number of studies have investigated the repeatability of activity levels and spatial patterns in fishes in natural settings, mainly based on acoustic telemetry. For example, Hanson *et al.* (2010) found that the daily distance travelled and mean daily swimming speed in largemouth bass *Micropterus salmoides* were repeatable on a daily basis, but not amongst seasons or across multiple winters. Working on a different species (*Salvelinus confluentus*), Taylor and Cooke (2014) found that the inter-individual movements were consistent across two seasons. More recently, Alós *et al.* (2017) found that activity levels and circadian rhythmicity (i.e. awaking time rest onset and rest duration) was repeatable for up to 14 days in the free-ranging pearly razor fish, *Xyrithchys novacula*. Harrison *et al.* (2015) found repeatable, cross-contextually consistent, personality-dependent home range, movement, dispersal from release and site fidelity (i.e. caused by individual differences in spatial use behaviours) over 2 years in *Lota lota*. Furthermore, Villegas-Ríos *et al.* (2017) found that diel vertical migration, activity, home range and dispersal of a marine species (Atlantic cod *Gadus morhua*) showed moderate to high individual repeatability in all behavioural traits.

However, common proxies for activity based on acoustic telemetry data proved to be poor descriptors of the activity levels (Pereñíguez *et al.*, 2022), at least for certain species that spend a large proportion of their time inside crevices and move without significant displacement, like the Patagonian grouper *A. patachonicus* (Ciancio *et al.*, 2016). The use of data loggers, including high-frequency tri-axial accelerometers, has provided a promising tool to monitor activity and behaviour of animals when they cannot be visually tracked (e.g. Murchie *et al.*, 2011; Kawabata *et al.*, 2014; Vázquez Diosdado *et al.*, 2015; Beltramino *et al.*, 2019; DeSantis *et al.*, 2020). This method also has the advantage that accelerometers can record large amounts of detailed information as they can register at frequencies of up to hundreds of Hertz (Hz). Additionally, accelerometers can be used as a proxy to monitor energy expenditure by relating animal activity with energy consumption (Wilson *et al.*, 2006, 2020)*.*

Although fish are particularly suited for behavioural studies employing tethered accelerometers, one of the mayor challenges is represented by the recovery of the external accelerometer. For this reason, most of the studies have been performed in mesocosms (Brownscombe *et al.*, 2014) or aquaria (Wright *et al.*, 2014), where the recovery of the loggers is highly reliable. In recent years, there has been a growing interest in the use of external accelerometers in the field. This is mainly due to the use of novel technologies and fishing techniques that increase the chances of recovering the tags and the collected data. Some examples include the development of tags that acoustically transmit averages or ranges of acceleration values (Murchie *et al.*, 2011; Carbonara *et al.*, 2021; Pereñíguez *et al.*, 2022; Lennox *et al.*, 2023), pop-off systems coupled with a VHF transmitter (Gleiss *et al.*, 2019) and also more specialized expertise of researchers to recapture the fish from the field and recover the tags (Gleiss *et al.*, 2013; Ciancio *et al.*, 2016; Beltramino *et al.*, 2019). This has led to an increasing number of studies using accelerometers to assess a diverse array of questions, such as the description of the post-release effects of fishing (Whitney *et al.*, 2016), the activity landscape for some species (Papastamatiou *et al.*, 2018), the swimming kinematics of large pelagic predators (Gleiss *et al.*, 2019) and the foraging strategies in billfish (Marras *et al.*, 2015). Despite the high-definition information they provide, accelerometers have been used infrequently to study repeatability of traits. Some examples include their use in free-living fishes (Rudd *et al.*, 2021), terrestrial mammals (Keegan *et al.*, 2011; Gottlieb *et al.*, 2013; MacKay *et al.*, 2014; Stiegler *et al.*, 2022) and birds (Grémillet *et al.*, 2018; Rotics *et al.*, 2021).

The Patagonian grouper *A. patachonicus* (Jenyns, 1840) is a sedentary, long-lived species that inhabits temperate rocky reefs in the southwest Atlantic Ocean (Irigoyen *et al.*, 2013). These fish are mostly observed in an upright body position outside reef crevices, up to a few metres above the seabed at near neutral buoyancy, or resting inside crevices at different roll angles (Ciancio *et al.*, 2016; Beltramino *et al.*, 2019). Previous studies showed that the activity budget for this species is modulated by water temperature and suggested that other environmental forcing variables (e.g. tide and ambient light levels) might have some effects at the individual level (Beltramino *et al.*, 2019). For example, some fish displayed a diel activity rhythm that closely followed ambient light levels, whilst the behaviour of other individuals was not so strongly affected by light (Beltramino *et al.*, 2019). Furthermore, this species has a high site fidelity, particularly during the warmer months when the Patagonian grouper is more abundant in shallow rocky reefs, and therefore has a high chance of recapture (Irigoyen, 2010). For these reasons, the Patagonian grouper was chosen as an appropriate model to study repeatability of activity traits in the field using accelerometer tags.

Here, we used external accelerometers to detect different swimming patterns (including hovering, swimming and other metrics related to tailbeat frequency) of the Patagonian grouper in the field in the short term (i.e. day-to-day and 1 week) within two periods (hereafter ‘seasons’ for simplicity): cold (July–October) and warm (December–May). We hypothesize that (1) swimming patterns are repeatable within the short term and (2) activity is higher during the warm season. This will provide insights on the repeatability of activity traits in marine fishes, as well as an assessment of the feasibility of accelerometry to explore inter-individual variability in activity in wild populations.

## Materials and Methods

### Animals and experimental protocol

**Figure 1:**
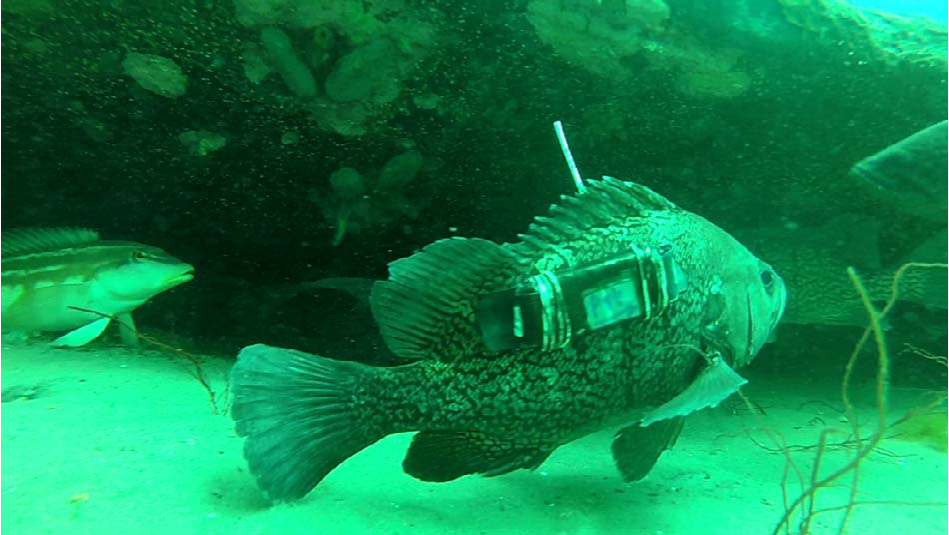
Patagonian grouper *A. patachonicus* tagged with an external accelerometer in the Golfo Nuevo, Argentina. Photograph by Lic. Agustín Biasotti (UNPSJB).

A total of 26 adult individuals of the Patagonian grouper, mean total length (TL) ± SD = 30.7 ± 3.6 cm, were captured between 2017 and 2019, on two reefs, at depths between 6.5 and 9.2 m, in the Golfo Nuevo, Northern Patagonia, Argentina (~43°S 65°W) (Supplementary Material S1). Fish were captured by scuba divers inside reef crevices by pole-hooking (Irigoyen and Venerus, 2008) or with a fish-landing net. After capture, fish were slowly brought to the surface in a dip net, and equipped with an accelerometer X16-mini (Gulf Coast Data Concept Company), with dimensions of 50 × 21 × 7 mm and mass of 17 g in air (Fig. 1). Accelerometers were encapsulated in heat-shrink tubes with enough air to achieve slight negative buoyancy of the whole package, and fixed to the right side of the body, at ~0.4 length, which is known to be near the centre of mass for teleosts (Webb, 1978), by using plastic straps or surgical thread. In both cases, we used surgical needles to pass the strap or the thread under the fish skin. The plastic straps were closed and the surgical thread was firmly knotted, in both cases by pressing against the heat-shrink tubes. The attachment procedure was preceded by spreading a solution of lidocaine 10% (Fullcaina, LAFEDAR) in the tagging area. In addition, the length of each fish was recorded and a spaghetti tag (Floy FD-68B) was attached at the base of the dorsal fin for individual identification. After a minimum of 8 days of free-living period, fish were recaptured using the same fishing technique and the accelerometers were removed from the fish. Fish were then gently released back into their crevices.

Mean water temperature in the area reaches a maximum in March and a minimum in September (Dellatorre *et al.*, 2012). We defined two sampling seasons based on mean water temperature: warm (*ca*.16–18°C) and cold (*ca*. 8–10°C). Tagging, undertaken in December, and from February to May, was considered representative of the warm season, whereas fish tagged from July to October were considered representative of the cold season.

### Ethical declaration

In Argentina there is not a legal instrument in place to ensure animal care during experimentation. However, the procedure for tagging and recapturing groupers was approved by the Institutional Animal Care and Use Committee (IACUC) from the CCT CONICET-CENPAT, Puerto Madryn, Chubut, Argentina.

### Data analysis

We used five dependent variables to explore repeatability in swimming activity of the Patagonian grouper; two of them (‘Swimming’ and ‘Hovering’), estimated by a classification algorithm, represent the percentage of time invested in swimming and hovering, respectively. The other three variables (‘Median tailbeat frequency’, ‘Percentage of high tailbeat frequency’ and ‘Total number of estimated tailbeats’) were based on proxies related to tailbeat frequency. The variables used are the following:

1) Swimming, defined as the percentage of time that fish spent swimming during a given day. We considered swimming as all the data points classified as such by a machine learning algorithm (see below). This can be considered as a proxy for when the fish was actively swimming at any speed, using the tail or the pectoral fins.
2) Hovering, defined as the percentage of time fish spent holding its position in the water column, during a given day. This was estimated using the data points classified as such by a classification algorithm (see below).
3) Median tailbeat frequency (MTBF), which was calculated as the median for each day, using a class width of 0.5 Hz, between 0.5 and 12 Hz (zero tailbeats were excluded from the calculation of the median). This lower limit is in line with previous work on fish of similar size, which yielded low speed ~0.6 length/s (L/s) or less, at ~1.2 Hz (Herskin and Steffensen, 1998; Steinhausen *et al.*, 2005; Ohlberger *et al.*, 2007). This is a proxy of how fast fish were swimming whilst active.
4) The percentage of high tailbeat frequency (i.e. >4 Hz) (HTB%) out of the total tailbeats per day. HTB is a proxy for swimming behaviour higher than sustainable. The threshold was based on previous work, which shows that maximum sustained speed for fish of similar size, is ~2–3 L/s (Videler, 1993). Using a stride length of 0.75 length (L), which is common in fish (Videler, 1993), this swimming speed corresponds to a tailbeat frequency ~2.6–4 Hz. Therefore, using a frequency of 4 Hz as a threshold should yield all events in which fish swam at a speed above their maximum sustained. This is an indication of how often fish are swimming in a burst-like mode whilst they were active.
5) The total number of estimated tailbeats (TTB) per day and individual, using 0.5 Hz as the minimum value as for MTBF. This is an indication of the activity level implying BCF swimming (Body Caudal Fin swimming *sensu*Webb, 1984) with tailbeat frequency (TBF) ≥0.5 Hz. This variable was log-transformed to deal with heteroscedasticity in the data (see Results).

Acceleration data were extracted from the accelerometers by using the procedure described in Beltramino *et al.* (2019). Although acceleration data were recorded for up to 14 days in a few individuals, a period of 8 days was dictated by the need of maximizing the number of fish and repeated days in the sample. Data from the first recording day (Day 1) were not included in the analysis to avoid potential manipulation effects and because they did not include a full day of recording. Similarly, the last day (Day 8) was not included because of the lack of a full day of recording in most cases. Hence, the longest period available for testing repeatability was from Day 2 to Day 7.

The details of the classification procedure are given in Supplementary Material S2. A training dataset, compiled from a total of seven fish (wild and captive) tagged with accelerometers and video recorded whilst performing different swimming activities, was used to classify the acceleration signals of the free-living fish into four categories selected for this species (details in Beltramino *et al.*, 2019). Amongst those categories, only ‘Swimming’ and ‘Hovering’, implying active movement rather than posture, were used in the present study to test repeatability in swimming activity in the Patagonian grouper. The daily percentage of time spent in each of these two activities was estimated by using the K-nearest neighbour classification algorithm (KNN, Bidder *et al.*, 2014). The other two categories, not used in this study, were ‘Static on bottom’, which implies that fish were resting on the bottom and movement was restricted, and ‘Lying on its side’, which includes fish resting at extreme roll angles (Ciancio *et al.*, 2016; Beltramino *et al.*, 2019).

To estimate a proxy for TBF, we split the ‘Sway’ raw acceleration (using data at 25 Hz for this analysis) into its static and dynamic components (Shepard *et al.*, 2008). Then, we used the *dfreq* function (2-s window with 50% overlap, and a 10% threshold) of the package ‘seewave’ (Sueur *et al.*, 2008) of the R software (R Core Team, 2020) to identify tailbeat cycles in the sway dynamic acceleration. The estimated TBF was validated in a swim tunnel, in which we swam tagged fish at controlled speeds (regression between the means of video-counted TBF and accelerometer-derived TBF: R^2^ = 0.76, *P* < 0.001, *n* = 19; unpublished data).

To perform all the analyses described, we coded *ad hoc* routines in R (R Core Team, 2020). The KNN classification requires the ‘class’ package (Venables and Ripley, 2002). To estimate the variances of roll and sway, we used the packages ‘xts’ (Ryan and Ulrich, 2023), ‘TTR’ (Ulrich, 2021) and ‘caTools’ (Tuszynski, 2021).

### Analyses of repeatability

The repeatability in the five activity variables ‘Swimming’, ‘Hovering’, ‘MTBF’, ‘HTB%’ and ‘TTB’ was tested using two different approaches. The first approach is based on the Spearman correlation coefficient (Marras *et al.*, 2010). This coefficient was used to test the repeatability of the daily value for each variable versus the same value for the successive day, e.g. Day 2 versus Day 3, Day 3 versus Day 4 and so on (Hanson *et al.*, 2010). We also focused on the repeatability on a weekly basis by comparing Day 2 versus Day 7, i.e. the longest time interval available. All Spearman coefficients were estimated in i) individuals tagged in the cold and in the warm seasons separately and ii) all individuals pooled together without considering the factor ‘Season’ (see below). *P*-values were adjusted for multiple comparisons amongst successive days for each variable and season (warm, cold and both) by using the Holm sequential correction (Holm, 1979) with the *p.adjust* function of the R software (R Core Team, 2020).

The second approach used to evaluate repeatability was based on the Intra-class Correlation Coefficient (ICC), which ranges from 0 to 1: it is 0 when all individuals have the same mean, and it is 1 when all individuals have different means but all measurements on the same individual are identical (i.e. perfect repeatability) (Becker, 1984; Lessells and Boag, 1987). ICC values were estimated from a series of repeated-measures models, fitted for each of the four activity variables under study. We used linear mixed-effects models because they present some advantages over other techniques (e.g. analysis of variance (ANOVA)); mainly, they do not hinge on either sphericity assumptions or complete data (Howell, 2009). In our study, e.g. the *dfreq* function failed to identify tailbeat cycles during Day 7 for one of the tagged fish during the cold season; hence one observation was missing for the variables ‘MTBF’, ‘HTB%’ and ‘TTB’ and an ANOVA approach was not feasible.

We fitted a series of models for each activity variable by including ‘Season’ with levels cold and warm, and ‘Day’, with levels Day 2,…Day 7, as fixed explanatory variables, TL as a covariate and ‘Fish identity’ (i.e. subject) as the random variable. Four models were fitted for each activity variable; all of them included the explanatory variables ‘Season’ and ‘Day’. In addition, two models included the covariate TL (with and without the interaction term ‘Season × Day’), and the other two did not include TL (with and without the interaction term ‘Season × Day’). Models were fitted by Maximum Likelihood (ML) (Zuur *et al.*, 2009) and were compared by using the Akaike Information Criterion, AIC (Akaike, 1973). For each activity variable, the model bearing the minimum AIC value was considered the best model. Those models differing by more than 2 in the AIC value with respect to that from the best model were discarded. The chosen models for each activity variable were refitted by restricted maximum likelihood (REML) before estimating ICC (Zuur *et al.*, 2009). A Gaussian error distribution with identity link was used for all models. The total number of tailbeats (‘TTB’) were log-transformed because data were clearly heteroscedastic. Diagnostic plots for the fitted models were obtained with the *simulateResiduals* function of the ‘DHARMa’ package (Hartig, 2022) of the R software (R Core Team, 2020).

We estimated the adjusted ICC (${ICC}_{adj}$), which represents repeatability that controls for confounding effects (Nakagawa and Schielzeth, 2010; Nakagawa *et al.*, 2017; Stoffel *et al.*, 2017), and is calculated by the expression:


$$ {ICC}_{adj}=\frac{\sigma_{\alpha}^2}{\sigma_{\alpha}^2+{\sigma}_{\varepsilon}^2} $$

where: ${\sigma}_{\alpha}^2$ is the variance of the random effect, which represents the between-subject variation, and ${\sigma}_{\varepsilon}^2$, the residual variance, which represents the within-subject variation. In addition, confidence intervals for repeatability were estimated by means of a parametric bootstrap (Stoffel *et al.*, 2017). We interpreted the ${ICC}_{adj}$ values following Liljequist *et al.* (2019), who suggested making qualitative interpretations of repeatability based on the categories: ‘poor’ (ICC < 0.5), ‘moderate’ (0.5–0.75), ‘good’ (0.75–0.9) and ‘excellent’ (ICC > 0.9).

We used the R packages ‘lme4’ (Bates *et al.*, 2015) and ‘rptR’ (Stoffel *et al.*, 2017) to fit linear mixed-effects models, and to estimate ${ICC}_{adj}$ values and their confidence intervals, respectively. Significance for statistical tests was set at *P* < 0.05 level.

**Figure 2:**
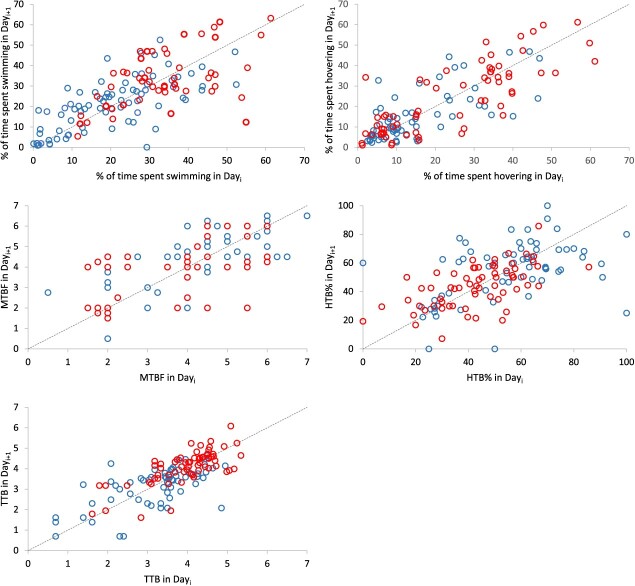
Relationship in daily percentage of time spent (a) swimming and (b) hovering, and in daily (c) median number of tailbeats (‘MTBF’), (d) percentage of high tailbeat frequency (‘HTB%’) and (e) logarithmized total number of tailbeats (‘TTB’), between two consecutive days, for the Patagonian grouper, discriminated by season. Open red circles: hot season; open blue circles: cold season. A bisector (dotted) line is included as reference in each plot.

## Results

**Figure 3:**
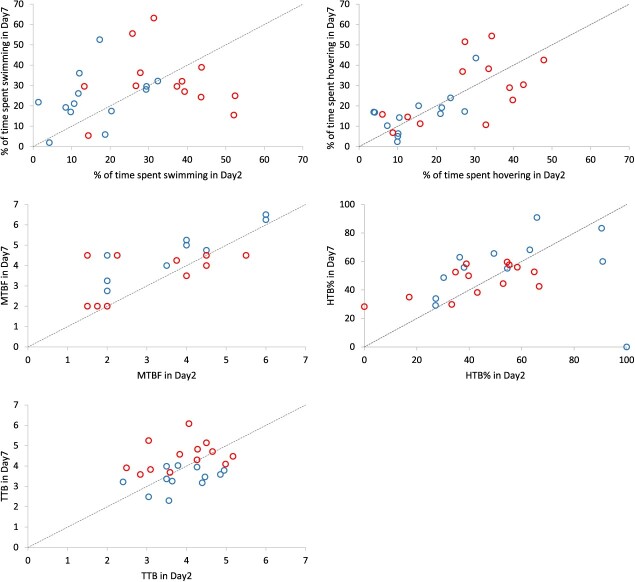
Relationship in daily percentage of time spent (a) swimming and (b) hovering, and in daily (c) median number of tailbeats (‘MTBF’), (d) percentage of high tailbeat frequency (‘HTB%’) and (e) logarithmized total number of tailbeats (‘TTB’), between the second and seventh day post-tagging, for the Patagonian grouper, discriminated by season. Open red circles: hot season; open blue circles: cold season. A bisector (dotted) line is included as reference in each plot.

All five dependent variables were significantly repeatable when Spearman correlations were analysed on a day-successive day basis for the pooled seasons, and in most cases when they were evaluated for each season separately, particularly for the warm season (Fig. 2 and Table 1). During that season, all correlations on a day-successive basis were significant for ‘Hovering’, ‘MTBF’ and ‘TTB’, and most were significant for ‘HTB%’ (four out of five). However, only two out of five correlations were significant for ‘Swimming’. During the cold season, all correlations on a day-successive basis were also significant for ‘Hovering’ and ‘TTB’, and most were significant for ‘Swimming’ (four out of five) and ‘MTBF’ (three out of five). The only exception to this general pattern was ‘HTB%’, which showed non-significant repeatability for most day-to-day pairings during the cold season (four out of five). Overall, this means that within the very short term (1 day), the swimming activity of fish was highly consistent. The Spearman correlations estimated on the longest time interval (Day 2 vs Day 7, i.e. weekly basis), were significant for four out of five variables: ‘Hovering’, ‘MTBF’, ‘HTB%’ and for ‘Swimming’, but in the latter case, only when both seasons were pooled together, probably due to the higher number of cases (Fig. 3 and Table 2).

**Table 1:** Results of the Spearman correlations between successive days for five dependent variables used to evaluate repeatability, based on acceleration data collected from 26 free-living Patagonian groupers tagged in the Golfo Nuevo, Península Valdés. The asterisks show significant correlations (*P* < 0.05)

Dependent variable	Warm season		Cold season		Both seasons pooled	
	rho (*n* = 13)	*P*	rho (*n* = 13)	*P*	rho (*n* = 26)	*P*
Swimming						
Day 2 vs Day 3	0.500	0.123	0.346	0.123	0.698	<0.001^*^
Day 3 vs Day 4	0.401	0.162	0.835	0.001^*^	0.832	<0.001^*^
Day 4 vs Day 5	0.692	0.022^*^	0.681	0.011^*^	0.765	<0.001^*^
Day 5 vs Day 6	0.412	0.162	0.703	0.011^*^	0.626	<0.001^*^
Day 6 vs Day 7	0.654	0.031^*^	0.775	0.004^*^	0.720	<0.001^*^
Hovering						
Day 2 vs Day 3	0.901	<0.001^*^	0.714	0.009^*^	0.791	<0.001^*^
Day 3 vs Day 4	0.923	<0.001^*^	0.775	0.004^*^	0.898	<0.001^*^
Day 4 vs Day 5	0.841	<0.001^*^	0.808	0.002^*^	0.856	<0.001^*^
Day 5 vs Day 6	0.549	0.026^*^	0.687	0.010^*^	0.628	<0.001^*^
Day 6 vs Day 7	0.901	<0.001^*^	0.599	0.015^*^	0.795	<0.001^*^
MTBF						
Day 2 vs Day 3	0.660	0.035^*^	0.400	0.175	0.569	0.001^*^
Day 3 vs Day 4	0.656	0.035^*^	0.729	0.011^*^	0.675	<0.001^*^
Day 4 vs Day 5	0.598	0.046^*^	0.628	0.043^*^	0.620	0.001^*^
Day 5 vs Day 6	0.514	0.046^*^	0.619	0.043^*^	0.680	<0.001^*^
Day 6 vs Day 7	0.561	0.046^*^	0.381 a)	0.175	0.610 b)	0.001^*^
HTB%						
Day 2 vs Day 3	0.659	0.021^*^	0.445	0.255	0.597	0.003^*^
Day 3 vs Day 4	0.451	0.061	0.643	0.044^*^	0.572	0.003^*^
Day 4 vs Day 5	0.583	0.036^*^	0.443	0.255	0.533	0.003^*^
Day 5 vs Day 6	0.697	0.016^*^	0.182	0.276	0.578	0.003^*^
Day 6 vs Day 7	0.726	0.012^*^	0.448 a)	0.255	0.685 b)	<0.001^*^
TTB						
Day 2 vs Day 3	0.648	0.017^*^	0.602	0.044^*^	0.650	<0.001^*^
Day 3 vs Day 4	0.747	0.007^*^	0.791	0.003^*^	0.818	<0.001^*^
Day 4 vs Day 5	0.927	<0.001^*^	0.553	0.044^*^	0.806	<0.001^*^
Day 5 vs Day 6	0.680	0.016^*^	0.774	0.004^*^	0.805	<0.001^*^
Day 6 vs Day 7	0.643	0.017^*^	0.622 a)	0.044^*^	0.802 b)	<0.001^*^

a) *n* = 12

b) *n* = 25

**Table 2:** Results of the Spearman correlations (between Days 2 and 7 post-tagging) for five dependent variables used to evaluate repeatability, based on acceleration data collected from 26 free-living Patagonian groupers tagged in the Golfo Nuevo, Península Valdés. The asterisks show significant correlations (*P* < 0.05)

Dependent variable	Warm season		Cold season		Both seasons pooled	
	rho (*n* = 13)	*P*	rho (*n* = 13)	*P*	rho (*n* = 26)	*P*
Swimming	−0.220	0.235	0.467	0.055	0.335	0.047^*^
Hovering	0.533	0.032^*^	0.632	0.012^*^	0.707	<0.001^*^
MTBF	0.502	0.040^*^	0.803	<0.001^*^	0.667	<0.001^*^
HTB%	0.527	0.032^*^	0.762 a)	0.002^*^	0.659 b)	<0.001^*^
TTB	0.368	0.120	0.352 a)	0.120	0.326 b)	0.056

a) *n* = 12

b) *n* = 25

**Figure 4:**
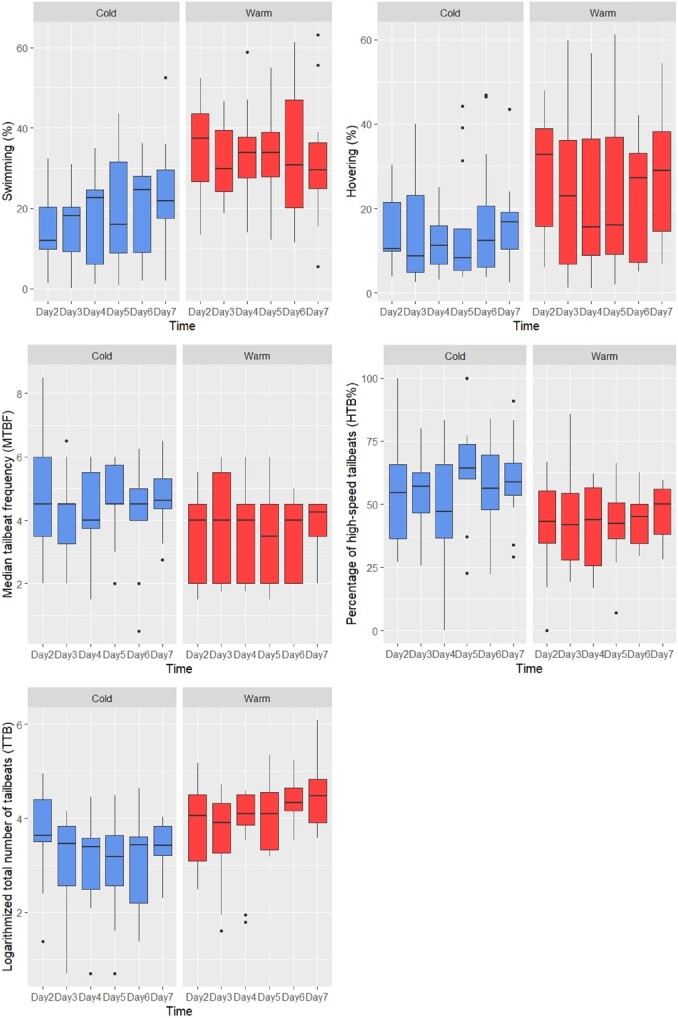
Data distribution for the five dependent variables studied: daily percentage of time spent (a) swimming and (b) hovering, and daily (c) median number of tailbeats (‘MTBF’), (d) percentage of high tailbeat frequency (‘HTB%’) and (e) logarithmized total numbers of tailbeats (‘TTB’), for the Patagonian grouper, discriminated by day and season.

The linear mixed-effects models for the variables ‘Swimming’, ‘Hovering’, ‘MTBF’, ‘HBT%’ and ‘TTB’ met normality and homoscedasticity assumptions (results not showed). In all cases, the models that incorporated TL as a covariate showed ΔAIC values between 1.7 and 1.9, with respect to the same models without TL, so that for parsimony, we finally chose the models that did not include the TL covariate. In the selected models for ‘Swimming’, ‘Hovering’, ‘MTB’ and ‘HTB%’, the interaction term ‘Season × Day’ did not reduce the residual deviance significantly so it was not retained. However, in the model for ‘TTB’, the interaction reduced the residual deviance significantly (ΔAIC = 6.17) and hence it was included, implying that the effect of the season varied through the days since tagging (Fig. 3). The percentage of time spent swimming or hovering was higher during the warm months: they increased 83.7% (*P* < 0.001) and 59.4% (*P* < 0.05), respectively, with respect to the cold period (Fig. 4). Conversely, the HTB% (>4 Hz) was 22.4% lower during the warm season (*P* < 0.05). There were no differences in the median number of tailbeats between seasons (*P* = 0.07); neither did we detect any effects of the day on any of the four variables: ‘Swimming’, ‘Hovering’, ‘MTBF’ or ‘HTB%’. The analysis of repeatability based on ${ICC}_{adj}$ showed that it was poor for ‘Swimming’ and ‘HTB%’ and moderate for the remaining three activity variables studied: ‘Hovering’, ‘MTBF’ and ‘TTB’ (Table 3).

## Discussion

Our results showed that most activity variables considered are repeatable in the short term (up to 1 week). We did not find large differences in the day-to-day repeatability between seasons. For each season, most variables showed significance in all, or in all but one or two, of the day-to-day pairings. The only exceptions to this general pattern were ‘HTB%’ and ‘Swimming’, for which most day-to-day pairings were weakly correlated during the cold and the warm seasons, respectively. When both seasons were pooled together, day-to-day pairings were significantly correlated for all the variables, possibly due to a greater statistical power resulting from the larger amount of data. The possibility that trait repeatability is maintained across different temperature regimes (i.e. cold vs warm seasons), beyond an increase in the average activity level during the warm season (particularly evidenced by the variables ‘Swimming’ and ‘Hovering’), is consistent with previous results by Claireaux *et al.* (2007), who found that sprint performance was repeatable after acclimatizing European seabass (*Dicentrarchus labrax*) at different temperatures (12 and 22°C), and with those by Hanson *et al.* (2010), who reported that both daily distance travelled and mean daily swimming speed in the largemouth bass (*M. salmoides*) were repeatable during most seasons. However, repeatability based on Spearman correlations on a week interval was higher in the cold season for four of the variables, ‘TTB’ being the only exception to this pattern, due to increasing values with days post-tagging. It is therefore possible that a general lower level of activity during the cold season diminishes the variability in activity, which may cause an increase in repeatability. Amongst the measured variables, ‘Hovering’, ‘MTBF’ and ‘TTB’ were the ones with the highest repeatability based on the ICC analysis. The repeatability of TTB suggests that the activity level (Body Caudal Fin locomotion, BCF *sensu*Webb, 1984) is individual-specific.

Whilst swimming in this species is mainly supported by the tail, hovering may also contribute importantly to oxygen consumption, as it was shown for a labriform fish (Tudorache *et al.*, 2009), and therefore to the overall energy budget. Individuals with a high hovering score may therefore have a higher daily energetic demand. The Patagonian grouper is a sedentary species that spends large periods in crevices to avoid predators, and it is also considered that this behaviour may reduce the costs of buoyancy control and posture maintenance (Ciancio *et al.*, 2016). Individuals that spend longer periods outside their crevices, hovering in the water column, may be more vulnerable to predation or fishing, which would lead to a selective removal from the reef (Lewin *et al.*, 2006). Therefore, the assessment of hovering repeatability may be valuable from a conservation viewpoint, as it would allow to predict which individuals are more likely to be caught repeatedly with potential effects on their overall survivorship. The selective removal of individuals with higher hovering scores in reefs subject to high fishing effort might modify the average energetic demand of fish at the local (i.e. reef) scale.

The high repeatability of ‘MTBF’ implies that individuals that swim faster than others did so day after day. Interestingly, ‘Swimming’ (which is a proxy for general activity per day) was less repeatable across the week period and it yielded the poorest correlation for both seasons pooled together, as well as the lowest ICC_adj_ amongst the variables tested, followed closely by ‘HTB%’ (both having an ICC_adj_ value slightly <0.5). This suggests that ‘MTBF’ is decoupled from the percentage of time spent in swimming. The HTB% > 4 Hz can be interpreted as the use of swimming speeds higher than sustainable, such as those commonly used in predator–prey interactions (Domenici and Blake, 1997). The repeatability of ‘MTBF’ and specifically that of ‘HTB%’ (though significant only for seasons pooled) suggests that certain individuals consistently perform more predatory attack or avoidance manoeuvres, as it is known that, e.g. high likelihood to respond to threat may be a personality trait (Jones and Godin, 2010). The Argentine sea bass tends to feed mainly on benthic prey that are not highly mobile such as polychaetes and crabs (Goldstein and Cousseau, 1987; Galván *et al.*, 2009), although they also opportunistically feed on pelagic fish such as anchovy (*Engraulis anchoita*). Therefore, their high-speed swimming activity recorded could be related to anti-predator behaviour but also, at least occasionally, to feeding strikes aimed at catching manoeuvrable fish in the water column.

The high repeatability found in the very short term (day-to-day) is in line with previous work on a freshwater species (Largemouth bass *M. salmoides*) based on activity level measured through whole-lake acoustic telemetry array with sub-metre accuracy (Hanson *et al.*, 2010). These authors found that the daily distance travelled and mean daily swimming speed was repeatable day-to-day within each season. A recent work also found repeatability in short (daily) and medium (monthly) time periods of locomotion kinematics and swimming performance variables for a Gymnotiform fish (Oufiero *et al.*, 2021). The repeatability of a similar trait (median swimming speed) has also been proved during the early stages of a clonal freshwater fish, *Poecilia formosa*, in periods up to 10 weeks (Laskowski *et al.*, 2022).

Accelerometers have proved to be useful tools to describe and quantify different aspects of animal behavioural ecology, with studies focused on the occurrence and intensity of behaviour, movement characteristics, body posture and energy expenditure (e.g. Ciancio *et al.*, 2016; Wilson *et al.*, 2020; Garde *et al.*, 2022; Lennox *et al.*, 2023). A few studies also focused on circadian rhythms (Alós *et al.*, 2017) and foraging patterns (Sala *et al.*, 2015), or evaluated how the behaviour of aquatic animals changes over time in response to changing environments (Stieglitz and Dujon, 2017). Admittedly, the primary current limitation of the present work is the restricted time frame, which is limited to a week interval. Previous work suggests that experiments performed at short time intervals tend to show higher repeatability than behaviours recorded at longer intervals (Bell *et al.*, 2009; Hanson *et al.*, 2010). Overall, telemetry studies on fishes strongly suggest the presence of repeatable activity patterns over relatively long periods in a number of species, although context-specific performances may change with differing environmental conditions across seasons and years (Hanson *et al.*, 2010). Therefore, it would be interesting to test if the activity traits that can be measured using accelerometry and are related to specific swimming patterns (e.g. hovering, swimming), show some degree of long-term (e.g. across seasons and years) repeatability. Although the need for long-term investigations based on accelerometry is challenging because of some technical issues such as the memory available and the size of the tag, new accelerometry tags currently under development will allow longer recording intervals in the near future (e.g. Lennox *et al.*, 2023).

**Table 3:** Adjusted ICCs (${ICC}_{adj}$ estimates) and their corresponding 95% confidence intervals (95% CI) for five dependent variables studied, based on acceleration data from 26 free-living Patagonian groupers tagged in the Golfo Nuevo, Peninsula Valdés

Dependent variable	${ICC}_{adj}$ estimate	95% CI
Swimming	0.403	[0.214–0.580]
Hovering	0.657	[0.473–0.787]
MTBF	0.547	[0.345–0.697]
HTB%	0.448	[0.261–0.640]
TTB	0.573	[0.388–0.727]

Compared to previous work based on other methodologies (e.g. ultrasonic telemetry, mark-recapture, observation, video recording, hormone titration, etc.), which have focused on studying the repeatability of activity levels, spatial pattern, boldness and stress levels (e.g. Cook *et al.*, 2011; Harrison *et al.*, 2015; White *et al.*, 2015; Villegas-Ríos *et al.*, 2017), the use of accelerometry can widen the spectrum of activities that can be monitored. Here, for example, we found that ‘Hovering’, an activity that cannot be detected via telemetry because it does not imply displacement, is repeatable within the short term (week). Similarly, accelerometry allows different swimming activities with relatively high resolution, such as low- versus high-speed swimming activity, to be detected, thus revealing how often fish are likely to be engaged in escape responses, mating or predator–prey-type interactions. Further work could investigate the possibility of partitioning burst-swimming activity into feeding versus anti-predator behaviour, as in laboratory studies (Broell *et al.*, 2013). On the other hand, certain fish traits such as home range and depth use are not detected by accelerometers, though they are detected by other methods such as acoustic telemetry. Therefore, the use of different techniques combined (e.g. Kawabata *et al.*, 2014; Meese and Lowe, 2020) is a promising avenue for monitoring the behaviour, the spatial patterns and the activity levels of fish, thereby providing a useful tool for assessing the inter-individual variability of fish populations. Maintaining inter-individual variation in movement patterns is important for a population’s ability to respond to future environmental change (Killen *et al.*, 2016). Hence, further research using accelerometry for measuring both the repeatability of behavioural traits and the effect of season and temperature on activity levels would be fundamental for assessing the resilience of fish populations to environmental disturbances, which can be considered a major advancement in the field of conservation physiology.

## Supplementary Material

Web_Material_coae074

## Data Availability

The data underlying this article will be shared on reasonable request to the corresponding author.
