# Supplementary material for: Repeatability of swimming activity of the Patagonian grouper Acanthistius patachonicus based on accelerometry
Source: Conserv Physiol. 2024 Oct 28;12(1):coae074. doi: 10.1093/conphys/coae074 (PMC11519044; doi:10.1093/conphys/coae074)
Supplement: Web_Material_coae074 [file web_material_coae074.zip › Suppl Mat _Repeatability Revised.pdf]

Repeatability of swimming activity of the Patagonian grouper *Acanthistius patachonicus* based on accelerometry

Leonardo A. Venerus[1*+], Paolo Domenici[2,3+], Stefano Marras[2], Lucas E. Beltramino[1] and Javier E. Ciancio[1]

[1] Centro para el Estudio de Sistemas Marinos, Consejo Nacional de Investigaciones Científicas y Técnicas (CESIMAR, CCT CONICET-CENPAT), U9120ACD Puerto Madryn, Chubut, Argentina.

[2] Consiglio Nazionale delle Ricerche, Istituto per lo studio degli impatti Antropici e Sostenibilità in ambiente marino (CNR-IAS), 09070 Torregrande, Oristano, Italy

[3] Consiglio Nazionale delle Ricerche, Istituto di Biofisica (CNR-IBF), 56124 Pisa, Italy

* **Corresponding author:** Centro para el Estudio de Sistemas Marinos, Consejo Nacional de Investigaciones Científicas y Técnicas (CESIMAR, CCT CONICET-CENPAT), Blvd. Brown 2915 (U9120ACD) Puerto Madryn, Chubut, Argentina. Phone: +54 (280) 488 3184 Ext. 1286. E-mail: leo@cenpat-conicet.gob.ar

[+] LAV and PD contributed equally to the elaboration of this work.

S1. Total length (in centimeters) of 26 individuals of Patagonian grouper *Acanthistius patachonicus* tagged with external accelerometers in two reefs located in the Golfo Nuevo, Northern Patagonia, Argentina: Punta Este and Parque Nuevo. Fish were tagged in December, and from February to May (warm season), and from July to October (cold season), between 2017 and 2019.

Season/Reef	Punta Este	Parque Nuevo
Warm	24, 27, 28, 28, 28, 29, 29. 30, 31, 31, 32, 37, 41	No fish were tagged
Cold	28, 29, 30, 30, 31, 32, 34, 35, 36	27, 29, 30, 31

S2. Classification procedure for the activity categories

To feed the classification algorithm we used the raw accelerometer data (measured in the three axis of the fish ‘Surge’, ‘Heave’ and ‘Sway’ [Wright *et al*., 2014]) and other derived variables: VeDBA (vector of the dynamic body acceleration, [Qasem *et al*. 2012]) and the variances of the raw sway (‘VariSway’) and roll (‘VariRoll’). A three-second running mean was used for estimating each of these variables. A training data set, compiled from a total of seven fish (wild and captive) tagged with accelerometers and video recorded while were performing the different swimming activities, was used to classify the acceleration signals of the free-living fish into four activity categories selected for this species: ‘Swimming’, ‘Hovering’, ‘Static on bottom’ and ‘Lying on its side’ (see details in Beltramino *et al*., 2019). Among those categories, only ‘Swimming’ and ‘Hovering’, that imply activity rather than posture, were used in the present study to test swimming activity repeatability in the Patagonian grouper. The daily percentage of time spent in each of these two activities was estimated by using the K-nearest neighbour classification algorithm (KNN, Bidder *et al*., 2014) with K = 3. Before running the classification, the dataset was subsampled from 25 to 1 Hz to reduce the temporal correlation between successive recordings and to decrease computational time. To avoid potential bias caused by the use of unbalanced and particular training data sets (i.e. training data sets composed by a different number of observations in each activity category), we constructed one hundred training data sets by randomly sampling 2983 observations for each activity category from the entire training dataset (that being the number of instances of ‘Hovering’, the least common category observed). The mode of the one-hundred classifications obtained for each observation was used as the classified activity. Then, daily percentage of time in each category was estimated for each fish. This procedure gave a reasonable macro-average F-measure (Özgür *et al*., 2005; Brewster *et al*., 2018) of 0.79 (in a 0-1 scale) for the KNN classification process, although ‘Swimming” and ‘Hovering’ showed lower F-measure values of 0.635 and 0.583, respectively (details in Table 1 Suppl. from Beltramino *et al*., 2019).

References

Beltramino LE, Venerus LA, Trobbiani GA, Wilson RP, Ciancio JE (2019) Activity budgets for the sedentary Argentine sea bass *Acanthistius patachonicus* inferred from accelerometer data loggers. *Austral Ecol* 44:397-408. https://doi.org/10.1111/aec.12696.

Bidder OR, Campbell HA, Gómez-Laich A, Urgé P, Walker J, Cai Y, Gao L, Quintana F, Wilson RP (2014) Love thy neighbour: automatic animal behavioural classification of acceleration data using the K nearest neighbour algorithm. *PLoS ONE* 9, e88609. https://doi.org/10.1371/journal.pone.0088609.

Brewster LR, Dale JJ, Guttridge TL, Gruber SH, Hansell AC, Elliot M, Cowx IG, Whitney NM, Gleiss AC (2018) Development and application of a machine learning algorithm for classification of elasmobranch behaviour from accelerometry data. *Mar Biol* 165, 62. https://doi.org/10.1007/s00227-018-3318-y.

Özgür A, Özgür L, Güngör T (2005) Text categorization with classbased and corpus-based keyword selection. Proceeding 20th Internat. Symposium on computer and information sciences (ISCIS, 2005). *Lect Notes in Comp Sci* 3733:606-615. https://doi.org/10.1007/11569596_63.

Qasem L, Cardew A, Wilson A, Griffiths I, Halsey LG, Shepard ELC, Gleiss AC, Wilson R (2012) Tri-axial dynamic acceleration as a proxy for animal energy expenditure; should we be summing values or calculating the vector? *PLoS ONE* 7, e31187. https://doi.org/10.1371/journal.pone.0031187.

Wright S, Metcalfe JD, Hetherington S, Wilson R (2014) Estimating activity-specific energy expenditure in a teleost fish, using accelerometer loggers. *Mar Ecol Prog Ser* 496:19-32. https://doi.org/10.3354/meps10528.
